# *NDUFV1* mutations in complex I deficiency: Case reports and review of symptoms

**DOI:** 10.1590/1678-4685-GMB-2021-0149

**Published:** 2021-11-19

**Authors:** Vanessa Zanette, Daniel do Valle, Bruno Augusto Telles, Alan J. Robinson, Vaneisse Monteiro, Mara Lucia S. F. Santos, Ricardo Lehtonen R. Souza, Cristiane Benincá

**Affiliations:** 1 Universidade Federal do Paraná, Departamento de Genética, Laboratório de Polimorfismos e Ligação, Curitiba, PR, Brazil.; 2 Hospital Pequeno Príncipe, Divisão de Neuropediatria, Curitiba, PR, Brazil.; 3 University of Cambridge, Medical Research Council, Mitochondrial Biology Unit, Cambridge, United Kingdom.

**Keywords:** Leigh Syndrome, mitochondrial diseases, metabolic acidosis, encephalomyopathies

## Abstract

Mitochondrial complex I (CI) deficiency is the most common oxidative phosphorylation disorder described. It shows a wide range of phenotypes with poor correlation within genotypes. Herein we expand the clinics and genetics of CI deficiency in the brazilian population by reporting three patients with pathogenic (c.640G>A, c.1268C>T, c.1207dupG) and likely pathogenic (c.766C>T) variants in the *NDUFV1* gene. We show the mutation c.766C>T associated with a childhood onset phenotype of hypotonia, muscle weakness, psychomotor regression, lethargy, dysphagia, and strabismus. Additionally, this mutation was found to be associated with headaches and exercise intolerance in adulthood. We also review reported pathogenic variants in *NDUFV1* highlighting the wide phenotypic heterogeneity in CI deficiency.

## Introduction

Dysfunctions in complex I (NADH ubiquinone dehydrogenase) (CI) represents a third of all early-onset mitochondrial disorders and are genetically and clinically diverse (Hirst, 2013). They are caused by mutations in mitochondrial and nuclear genes, with clinical phenotypes ranging from severe lactic acidosis and death in infants to muscle weakness in adults. Five clinical groups were associated with CI deﬁciency: Leigh syndrome, progressive leukoencephalopathy, neonatal cardiomyopathy, severe infantile lactic acidosis, and a miscellaneous group of unspeciﬁed encephalomyopathies (Fassone and Rahman, 2012). Correlations between genotype and phenotype in mitochondrial diseases are not fully understood and need further exploration (Baertling *et al.*, 2018)

CI is a protein complex composed of 44 different subunits, seven of which are encoded by mitochondrial DNA (mtDNA) and the remaining by nuclear DNA (nDNA) (Hirst, 2013). CI catalyzes the oxidation of nicotinamide adenine dinucleotide (NADH) in the mitochondrial matrix, supplying electrons to complex III via reduction of coenzyme Q. The electron flux through CI sustains the translocation of four protons across the inner mitochondrial membrane, thus contributing to the mitochondrial electrochemical potential. CI is also an important source of reactive oxygen species (ROS) in mitochondria, which causes cellular oxidative stress (Murphy, 2009). CI is subdivided into three modules: the electron input (N) module, the electron output (Q) module, and the proton translocations (P) module. The N module is composed of subunits encoded by the genes *NDUFV1*, *NDUFV2*, and *NDUFS1* (Brandt, 2006)*.* The *NDUFV1* gene (NG_013353.1) is located on chromosome 11 and encodes the NADH-ubiquinone oxidoreductase flavoprotein (NM_007103.4), a hydrophilic polypeptide of 51 kDa, which oxidizes NADH and is a major site of ROS production (Hirst, 2013; Varghese *et al.*, 2015).

In this report, we presented three patients with mutations in the *NDUFV1* gene. The first patient is a compound heterozygous for a missense mutation c.640G>A (p.Glu214Lys) and a frameshift mutation c.1207dupG (p.Asp403Gly*fs), the second is homozygous for a missense mutation c.1268C>T (p.Thr423Met), and the third, combining the c.1268C>T (p.Thr423Met) and a likely pathogenic variant c.766C>T (p.Arg256Cys).

## Methods

This work received the approval of the ethical standards committee of the Federal University of Paraná (CAAE: 84773818.2.0000.0102).

Total DNA was extracted from blood using the DNeasy Kit (Qiagen, Hilden, DE). Whole Exome Sequencing (WES) was carried out using Illumina® 2000 HiSeq with Agilent SureSelect Human All Exon V7 for the first two cases, and Nextera® Exome Capture to third, the GRCh37 reference genome and a GATK-based pipeline was used to call, filter and annotate variants (Van der Auwera *et al.*, 2013). After identification of relevant variants, the region containing each variant was re-sequenced and family segregation was performed by Sanger sequencing.

The position of the mutations was mapped to the corresponding amino acids in the protein structures of *Bos taurus* (NM_174808.1) and human (PDB ID: 5XTB). The impact of the mutated amino acids on CI was investigated by visualizing the mutations in the context of the CI structures (Zhu *et al.*, 2016) by using the PyMOL structure viewer.

MR imaging and MR spectroscopy of the brain were performed with a 1.5-T MR unit (GE Medical Systems, Milwaukee, WI). T1-weighted images [echo time (TE)/repetition time (TR) 11 ms/550 ms], T2-weighted images (TE/TR 93 ms/4000 ms), fluid-attenuated inversion recovery (FLAIR) (TE/TR/inversion time 110 ms/10000 ms/2250 ms) and diffusion-weighted images (DWI) (TE/TR: 105 ms/5200 ms) were performed. Spectroscopic imaging was performed with long (144 ms) and short TE (35 ms). 

The pathogenicity was predict by SNPs&GO (Calabrese *et al.*, 2009), PolyPhen-2 (Ramensky *et al.*, 2002) e MutationTaster (Schwarz *et al.*, 2014) and allele frequency was searched in Exome Aggregation Consortium (EXAC) (Lek *et al.* 2016), 1000genomes (Consortium T 1000 GP, 2015), gnomAD (Karczewski *et al.*, 2020) AbraOM (Naslavsky *et al.*, 2017), TopMed (Taliun *et al.*, 2021) and Kaviar (Glusman *et al.*, 2011). Association between mutation c.766C>T and symptoms in family members of patient P3 were estimated by Chi-Square test with Monte Carlo simulations by using the R program.

## Results

### Case reports

*Patient P1*

Patient 1 (P1), male, 4 years old, was born by cesarean delivery at 40 weeks of gestation with 3760 g. The mother presented three previous miscarriages (8-, 8- and 12-weeks’ gestation) and placenta detachment during P1 pregnancy with indication of resting. The father presented childhood seizures and no consanguinity was reported. P1 had three healthy brothers, two of whom were maternal half-brothers.

P1 was 3 years old when he presented developmental delay and autistic spectrum disorder and was admitted to the hospital after episodic vomiting and dehydration associated with a cutaneous rash. After treatment P1 developed respiratory insufficiency needing orotracheal intubation. Viral encephalitis was evaluated with negative outcomes in cerebrospinal fluid (CSF), however empiric treatment with Acyclovir for 14 days showed complete recovery of neurological symptoms, with normal daily activities and no functional impairment. Brain MRI was performed after treatment and showed T2-weighted hyperintense and T1-weighted hypointense focal lesions in central portions of the upper segment of the cervical spinal cord, with additional symmetric lesions in midbrain, pons, and bulb. 

**Figure 1 -:**
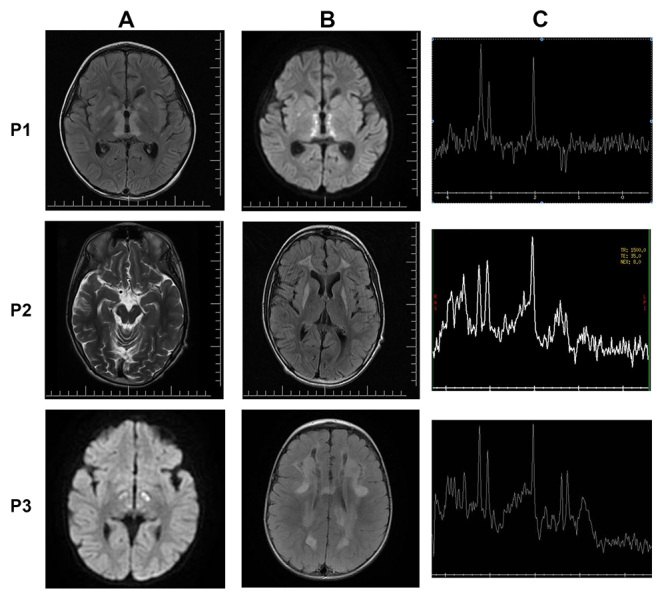
Clinical features of the patients. (A and B) MRI of patients P1, P2 and P3 showing white matter changes and symmetric lesions in different regions of the brain, red arrow indicated altered brain region. (C) Elevated lactate in each patient measured by MR spectroscopy, indicated by red arrow.

One month later, P1 was admitted to the hospital with prostration, nausea, vomiting and absence seizures, developing a cutaneous rash and apnea after two days. Orotracheal intubation was performed and extubation failure occurred due to the persistency of apnea, therefore, tracheostomy and gastrostomy were performed. Arterial blood gas analysis, blood lactate, blood acylcarnitine’s profile, urinary organic acids, and blood amino acid chromatography were normal. The patient continued to have recurrent apneas, but no seizures. A new MRI showed increased hyperintense lesions in T2 and hypointense lesions in T1, affecting the central portions of the upper segment of the cervical spinal cord (Figure 1, P1: A-B). Symmetric lesions also increased when compared with the first MRI, mainly affecting the mesencephalon, pons, medulla oblongata, cerebellum and upper portions of the cervical spinal cord with an elevation of lactate detected by MR spectroscopy (Figure 1, P1: C). 

Based on the hypothesis of mitochondrial disease, treatment was started with coenzyme Q10 (CoQ) (300 mg/day) and L-carnitine (100 mg/kg/day). At 4 years old, P1 presented slight clinical improvement with apnea stabilization, progressive drowsiness reduction and progress in wakefulness. 

*Patient P2*

Patient 2 (P2), male, 11 years old, was born by cesarean delivery at 38 weeks of gestation with 2470 g, 45 cm height and 33.5 cm head circumference. Pregnancy was uneventful and no symptoms were reported in the first four months of life. The mother was 46 years old when pregnant and presented one previous miscarriage. P2 had a deceased 5 years old sister with a clinical history of seizures, and a healthy older sister. Parents were consanguineous (first degree cousins).

At 4 months old, P2 presented minor cervical hypotonia and mild right hemibody hypertonia. At 7 years of age, he started to present developmental delay, aggressive behavior, learning difficulties, and motor regression. An organic acid profile in urine showed 3-methylglutaconic aciduria. He was admitted to hospital in the same year due to lethargy alternated with irritability and needed orotracheal intubation with mechanical ventilation after apnea. Tracheostomy was performed due to the extubation failure.

When P2 was 11 years old, he presented optic atrophy, sensorineural deafness, ptosis, hypotonia, hyperreflexia with diplegic spasticity, dysphagia and hyperhidrosis. Similar brain MRI patterns were seen in P2 at the age of 7 (Figure 1, P2: A) and 10 (Figure 1, P2: B) years. The second MRI showed hyperintensity in T2/FLAIR/T2 in the thalamus, lentiform nucleus, frontal lobe, and a lactate peak at spectroscopy (Figure 1, P2: C). Current treatment includes CoQ (300 mg/day), L-carnitine (2 g/day), carbamazepine (17.5 mg/kg/day), biotin (20 mg/day), creatine (2 g/day), clobazam (0.4 mg/kg/day), baclofen (15 mg/day), vitamin C and B complex and P2 is in a stable clinical condition.

*Patient P3*

Patient 3 (P3), female, 3 years old, the younger of two siblings from non-consanguineous parents. The mother had 4 pregnancies, with one miscarriage at 10 weeks. P3 had uneventful perinatal history, she was born at term, and had no clinical symptoms. Neurodevelopment milestones were normal up to 9 months old when she developed progressive hypotonia and somnolence, followed by a gradual loss of motor skills in the following month. Cerebrospinal fluid was negative for markers of viral infection. Her sister was healthy, however, her father presented epilepsy in infancy and frequent headaches in adulthood.

The first diagnostic hypothesis for P3 was acute disseminated encephalitis, showing normal electroencephalogram, diffuse white matter lesions by MRI and a lactate peak on spectroscopy (Figure 1, P3: A-C). Symptoms improved after treatment with methylprednisolone for three days, with an improvement of the symptoms. However, after a week, a gradual clinical decline was observed with lethargy, dysphagia and general hypotonia. After 13 months, a new MRI (not shown) showed increased white matter lesions, suggesting a mitochondrial disorder. An acylcarnitine profile in blood showed increased concentrations of 3-hydroxy-butyrylcarnitine and acetylcarnitine, amino acid chromatography showed increased alanine (656.1, normal 146 - 494 mcmol/L). Lactate levels in plasma ranged from 2.7 to 7.6 (normal <2.2mmol/L) over one year. Lactic acid levels in cerebrospinal fluid were often increased, ranging from 3.5 to 5.3 (normal <0.8mmol/L). Levels of urinary organic acids were normal. Supplementation with CoQ and carnitine was started but with no significative improvement. At 3 years old, P3 lost neurodevelopmental milestones and developed hypotonia, strabismus and severe inappetence. She was submitted to an endoscopic gastrostomy and current treatment was based on supplementation with CoQ, creatine, sodium bicarbonate, acetyl L-carnitine, riboflavin, and biotin.

### Genetic analysis

P1 revealed the heterozygous mutations c.640G>A (p.Glu214Lys) of maternal inheritance and c.1207dupG (p.Asp403Glyfs*27) from paternal inheritance in *NDUFV1* gene. The allele frequency of p.Glu214Lys was not reported, whereas p.Asp403Glyfs*27 shows a frequency of 0.0008% (ExAC and gnomAD) and 0.0004 (TOPMED) in control population, the frameshift mutation causes the premature stop codon of the protein in Asp429.

**Figure 2 -:**
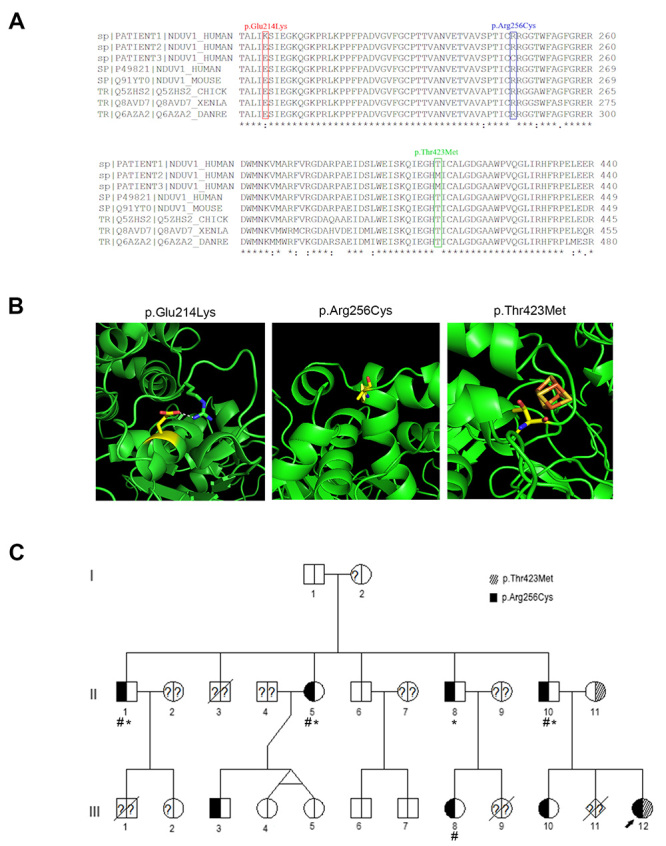
NDUFV1 protein and mutations. A) Sequence alignment of the NDUFV1 protein from different species. Mutated amino acids were indicated (patient 1:p.Glu214Lys; patient 2: p.Thr423Met; and patient 3: p.Arg256Cys); B) Representation of 3D structure of amino acid changes (p.Thr423Met , p.Arg256Cys and p.Glu214Lys); C) Pedigree of patient 3 (indicated by arrow) and family members affected by mutations c.766 C> T and c.1268C> T in the *NDUFV1* gene. Carriers of c.766 C> T present frequent headaches (#) and mild exercise intolerance (*).

The Glu214 residue was highly conserved among bilaterally symmetrical metazoans (Figure 2A) and was positioned in the interface with the subunit NDUFS4 linked by a hydrogen-bonded (Figure 2B). The mutation p.Glu214Lys disrupted the hydrogen bond and likely destabilized the interface with the neighboring subunit, NDUFS4.

P2 shows the c.1268C>T (p.Thr423Met) mutation in homozygosity in the *NDUFV1* gene. The highest allele frequency in control population was below 0.01%. The Thr423 residue was highly conserved among bilaterally symmetrical metazoans (Figure 2A) and faces the 4Fe-4S domain of NDUFV1 (Figure 2B). The mutation of threonine to a larger methionine was likely to disturb the assembly of the 4Fe-4S cluster into the protein or to cause dysfunction in the assembled protein.

WES analysis of P3 identified the compound heterozygous mutations in the *NDUFV1* gene, c.766C>T (p.Arg256Cys) and c.1268C>T (p.Thr423Met). Analysis of the mutations in the family members showed c.766C>T segregating from the father, and c.1268C>T from the mother (Figure 2C). Five members of the paternal lineage were carriers for c.766C>T. The mutation c.766C>T caused the missense change p.Arg256Cys with no associated clinical conditions. Allele frequency of this variant corresponded to one heterozygous individual (0.00001648%) only and bioinformatic tools predicted this alteration as pathogenic (score 0.99 and 0.87). The amino acid arginine in this position was highly conserved in bilaterally symmetrical metazoans (Figure 2A). Arg256 was localized in the tip of the hydrophilic arm of NDUFV1 subunit and this sidechain was not resolved in the protein structure (Figure 2B). Family carriers for p.Arg256Cys presented frequent headaches and exercise intolerance (Figure 2C) with statistically significant (p-value 0.027) association between frequent headaches and the mutation was found.

### Literature review

Several studies described mutations in the *NDUFV1* gene associated with CI deficiency (Table 1). In this review we found 24 patients with mutations in the *NDUFV1* gene, of which 8 were homozygous and 15 were compound heterozygous. The most frequent mutations were c.1268C>T (p.Thr423Met) and c.1156C>T (p.Arg386Cys).

The onset of symptoms ranged from 5 months of age to adulthood. Several common symptoms were described, mainly neuromotor regression, with spasticity, tremors, ataxia, dystonia, seizures, hypotonia, apnea, dysphagia, vomiting, lethargy, ptosis, strabismus, nystagmus, optic atrophy, cognitive decline, autistic behaviors, progressive weakness and exercise intolerance.

The most common features in MRI were hyperintense lesions in T2 affecting several regions of the brain, white matter changes, brain atrophy, and presence of lactate peak in MR spectroscopy.

**Table 1 -:** Clinical and molecular features of patients presenting *NDUFV1* mutations. Nucleotide and amino acid change, clinical phenotype, brain MRI, Het/Hom (Heterozygous or homozygous), segregation and references are described. C.het. (Compound Heterozygous); Hom (Homozygous); Mat. (maternal); Pat. (Paternal); Ref (Reference). Lines in blue: Cases of p.Thr423Met, lines in green: cases of p.Glu214Cys.

Nucleotide change	Amino acid change	Clinical phenotype (age of onset)	Brain MRI	Het/Hom	Segregation	Ref
c.640G>A c.1207dupG	p.Glu214Lys p.Asp403Gly^*^fs	Developmental/cognitive delay, autistic behaviors, vomits, apnea (3 y/o)	Hyperintense signal in T2, hypointense in T1 affecting the central portions of the upper segment of the cervical spinal cord. Symmetric lesions affecting the mesencephalon, bridge, bulb, cerebellum. Lactate peak	C. het	Mat. c.640G>A Pat. c.1207dupG	Present Report
c.1268C>T	p.Thr423Met	Cervical hypotonia, right hypertonia, lethargy, apnea (1 y/o) Optic atrophy, sensorineuronal deafness, ptosis, global hypotonia, hyperreflexia with diplegic spasticity, dysphagia and hyperhidrosis (11 y/o)	Hyperintensity in T2/FLAIR in thalamus, lentiform nucleus, frontal lobe, corpus callosum knee. Lactate peak	Hom	Both	Present Report
c.1268C>T c.766C>T	p.Thr423Met p.Arg256Cys	Hypotonia, lethargy, motor regression and dysphagia (10 m/o), neurodevelopmental delay and strabismus (2 y/o)	Diffuse white matter lesions and lactate peak	C. het	Mat. c.1268C>T Pat. c.766C>T	Present Report
c.766C>T	p.Arg256Cys	Infant seizures, exercise intolerance and frequent headaches (adulthood)	N/A	Het	N/A	Present Report
c.1268C>T	p.Thr423Met	Myopathy, depression, fatigue (infancy)	Normal	Het	Maternal	Baertling *et al.*, 2018
c.1268C>T	p.Thr423Met	Horizontal nystagmus, dysarthria, bilateral dysmetria and intention tremor, dysdiadochokinesia, and gait ataxia (10 y/o)	Bilateral, symmetric, hyperintense signal in the putamen and right caudate nucleus on T2-weighted imaging and a high lactate peak in the affected areas	Hom	Both	Incecik *et al.*, 2018
c.1118T > C	p.Phe73Ser	CI deficiency symptoms N/D (6 m/o)	N/D	Hom	Both	Srivasta *et al.*, 2018
c.1156C>T	p.Arg386Cys	Neuroregression, mild cognitive decline with regressive speech deficiencies, bilateral optic atrophy, and marked motor decline (6 y/o)	Diffuse white matter demyelination with cystic areas consistent with neurodegeneration	Hom	Both	Srivasta *et al.*, 2018
c.1156C>T c.155+1G>G/A	p.Arg386Cys	Spasticity of all four limbs, brisk deep tendon reflexes and extensor plantar response, Ophthalmic evaluation revealed bilateral horizontal nystagmus with normal optic disc (1 y/o)	Diffuse cystic leukoencephalopathy involving corpus callosum, deep and periventricular white matter with sparing of basal ganglia, brainstem, and cerebellum	C. het	N/D	Wadhwa *et al.*, 2018
c.605C>T c.816T>G	p.Ala202Val p.His272Gln	Dystonia, increased muscle tension, MR, left ventricular high voltage (21 m/o)	abnormal signals in bilateral Basal ganglia, brain stem and thalamus	C. het	Mat c.605C>T Pat.c.816T>G	Fang *et al.*, 2017
c.1162+4A>C c.640G>A	p.Gly214Lys	Seizures, ptosis, scoliosis, dystonia, (2 y/o)	Symmetrical putaminal lesions, lactate peak, involvement of the left, body of the caudate and the right quadrigeminal plate	C. het	Pat. c.1162+4A>C Mat. c.640G>A	Nafisinia *et al.*, 2016
c.365C>T c.158T>C	p.Pro122Leu p.Leu53Pro	Progressive weakness, epileptic seizures, optic atrophy, nystagmus, energy deficiency intolerance, learning disability (6 m/o)	white matter changes with leukomalacia and thinning of the corpus callosum, sparing the basal ganglia	C. het	Mat. c.365C>T Pat. c.158T>C	Bjorkman *et al.*, 2015
c.365C>T c.158T>C	p.Pro122Leu p.Leu53Pro	Irritability, progressive weakness, epileptic seizures, optic atrophy, strabismus, energy deficiency intolerance, learning disability (7 m/o)	progressive white matter changes with cystic malacic degeneration	C. het	Mat. c.365C>T Pat. c.158T>C	Bjorkman *et al.*, 2015
c.1156C>T c.914-8G_947	p.Arg386Cys	Inability to sit, poor head control, spasticity, brisk reflexes, sustained clonus, strabismus and nystagmus (1 y/o)	extensive atrophy of the white matter	C. het	Pat.r c.1156C>T Mat. c.914-8G_947	Recalde *et al.*, 2013
c.1156C>T	p.Arg386Cys	Mild titubation, drooling, increased irritability, axial hypotonia, lower extremity hypertonia, diffusely brisk reflexes and an extensor plantar response bilaterally with gait ataxia and frequent falls (14 m/o)	Bilateral symmetric hyperintense signal on T2-weighted imaging in periventricular white matter, centrum semiovale, corpus callosum, substantia nigra and periaqueductal gray associated with cystic necrosis, with a high lactate peak, decreased N-acetylaspartic acid (NAA) peak and increased choline peak	Hom	Both	Marin *et al.*, 2013
c.1156C>T	p.Arg386Cys	Developmental regression with intercurrent illness, left eye esotropia, dysphagia , ataxia, left hemi-body dystonic posturing, generalized spasticity, diffusely brisk reflexes, and extensor plantar responses (1 y/o)	N/D	Hom	Both	Marin *et al.*, 2013
c.262C>G c.596G>C	p.Arg88Gly p.Arg199Pro	Hypotonia, decreased spontaneous movements and hyperreflexia in the left lower extremity (32 m/o)	Symmetrical restricted diffusion of the corticospinal tracts and a lactate peak on MRS in the basal ganglia, thalamus and cortex	C. het	N/D	Marin *et al.*, 2013
c.1156C>T c.753delCCCC	p.Arg386Cys p.Ser251Ser^*^fs	Becoming non-verbal, unable to sit, irritability, horizontal nystagmus, dysphagia, tremor, upper extremity weakness, axial hypotonia with appendicular hypertonia, hyperreflexia, and extensor plantar responses bilaterally (14.5 m/o) Decreased visual acuity, dysphagia and complex partial seizures (7 y/o)	MRIs initially showed an improvement in the white matter signal changes and new areas of restricted diffusion within the frontal lobes but later revealed increases in the abnormal whitematter signals with newinvolvement of the basal ganglia, diffuse cystic change and an elevated lactate peak in the basal ganglia	C. het	N/D	Marin *et al.*, 2013
c.G1156A	p.Arg386His	Recurrent vomiting, dysphagia and fail- ure to thrive, axial hypotonia, tetraparesis without mus- cle wasting, irritability, and a rotatory nystagmus. hypoventilation of increasing intensity and rapid neurologic degradation (3.5 m/o)	T2 hypersig- nal and T1 hyposignal in the posterior part of the medulla, the pons and in the mesencephalon	Hom	Both	Vilain *et al.*, 2012
c.G1156A	p.Arg386His	rotatory nystagmus and mild peripheral hypotonia, Feeding difficulties and res- piratory insufficiency (3.5 m/o)	discrete symmetric T2 hyper- signal and T1 hyposignal lesions in the pons and the medulla	Hom	Both	Vilain *et al.*, 2012
c.770G>A c.632T>C	p.Arg257Gln p.Ala211Val	Regression in motor milestones stopped crawling and sitting independently (9 m/o)	Periventricular white matter abnormalities with sparing of the subcortical white matter	C. het	N/D	Zafeiriou *et al.*, 2008
c.640G>A c.1192+4A>C	p.Glu214Lys	Seizures (1 y/o), Cerebellar ataxia, psychomotor regression, strabismus and ptosis (28 m/o)	Brain atrophy and multiple symetric areas of hyperintensity in brain stem	C. het	Pat. c.640G>A Mat. c.1192+4A>C	Benit *et al.*, 2001
c.1294G>C	Ala432Pro	Vomiting, hypotonia, lethargy and apnea (6 m/o)	areas of hyperintensity in the basal ganglia.	C. het	Pat. c.1294G>C Mat. c.990_991del	Benit *et al.*, 2001
c.611A>G c.616T>G	p.Tyr204Cys p.Cys206Gly	Hypotonia, unable to sit, ataxia, bilateral ptosis and ophthalmoplegia (5 m/o)	areas of hyperintensity of the locus niger	C. het	Pat. c.611A>G Mat. c.616T>G	Benit *et al.*, 2001
c.175C>T c.1268C>T	p.Arg59X p.Thr423Met	Strabismus, progressive muscular hypotonia, myoclonic epilepsy and psychomotor regression. (5 m/o.)	cranial MRIs nor post-mortem reports were available to confirm symmetric midbrain or brainstem necrosis to definitively confirm Leigh syndrome	C. het	Pat. c.175C>T Mat. c.1268C>T	Schuelke *et al.*, 1999
c.1022C>T	Ala341Val	infantile myoclonic epilepsy, spasticity (6 m/o)	brain atrophy and a progressive macrocystic leukodystrophy	Hom	Both	Schuelke *et al.*, 1999
c.1118C>T	p.Phe373Ser	Seizures, myopia, bilateral lower set ears, nystagmus, mosaic pigmentary anomalies, hepatomegaly, and spasticity in lower limbs, extreme plantar responses and brisk deep tendon reflexes (6 m/o)	diffuse hyperintensity in the cerebral white matter, cerebellar white matter and brainstem white matter, and small cystic areas in the periventricular white matter	Hom	Both	Schuelke *et al.*, 1999

## Discussion

This work presents three patients with clinical presentation of mitochondrial disease with mutations in the *NDUFV1* gene (Table 2). The mutation p.Asp403Glyfs*27 was found in trans with p.Glu214Lys in P1, and the combination of p.Arg256Cys with p.Thr423Met was observed in P3. Both p.Glu214Lys and p.Thr423Met were described as pathogenic and cause CI deficiency (Baertling *et al.*, 2018; Incecik *et al.*, 2018; Srivastava *et al.*, 2018; Wadhwa *et al.*, 2018). The homozygous mutation p.Thr423Met in *NDUFV1* gene was found in P2.

The mutation p.Asp403Glyfs*27 leads to a truncated protein with likely functional consequences due to a stop codon preceding the 4Fe-4S domain (Hirst, 2013), and was not previously associated with mitochondrial disease. Despite this, the frameshift variant should be classified as pathogenic following the ACMG guideline. The variant p.Glu214Lys was previously described as likely pathogenic in a patient with seizures, cerebellar ataxia, and psychomotor regression presenting CI deficiency (Incecik *et al.*, 2018). The phenotype described was different from P1, which presented a mitochondrial disease phenotype characterized by developmental delay, autistic spectrum disorder, respiratory insufficiency, T1-weighted hypointense, symmetric lesions in white matter and presence of lactate peak at spectroscopy. The recurrent apneas, prostation and vomiting led P1 to hospitalization several times, making artificial respiration necessary, despite this, metabolic and laboratory profiles were normal.

**Table 2 -:** Classification of the variants identified in P1, P2, and P3 according to the ACMG criteria.

Patient	Location (Chromossome 11)	Variant NM_007103.4	Zygosity	**Classification (*ACMG*)**	Inheritance
*P1*	*67377981*	c.640G>A (p.Glu214Lys)	Heterozygous	Pathogenic (PS1+PM1+PM2+PM3)	Maternal
	*67379629*	c.1207dupG (p.Asp403Glyfs^*^27)	Heterozygous	Pathogenic (PVS1+PM2+PM3)	Paternal
*P2*	*67379696*	c.1268C>T (p.Thr423Met)	Homozygous	Pathogenic (PS1+PS3+PM2)	Both
*P3*	*67379696*	c.1268C>T (p.Thr423Met)	Comp. Heterozygous	Pathogenic (PS1+PS3+PM2)	Maternal
	*67378531*	c.766C>T (p.Arg256Cys)	Comp. Heterozygous	Likely Pathogenic (PM2+PM3+PP3+PP4)	Paternal

p.Glu214Lys changed a medium-sized acid residue to a large basic amino acid and may prevent hydrogen bonds, thus destabilizing the interface with the subunit NDUFS4. Mutations in this position probably reduce electron transfer through the redox Fe-S centers of CI (Incecik *et al.*, 2018). Modeling of the reported mutation in NDUFV1 suggested a disturbance of the assembly or function of the 4Fe-4S cluster (Fang *et al.*, 2017)

P2 was identified as homozygous for c.1268C>T (p.Thr423Met) mutation in *NUDFV1*. This mutation was described leading to myopathy, depression, fatigue, strabismus, progressive muscular hypotonia, myoclonic epilepsy and psychomotor regression. Another case of homozygosity for c.1268C>T was described leading to horizontal nystagmus, dysarthria, bilateral dysmetria and intention tremor, dysdiadochokinesia, and gait ataxia (Wadhwa *et al.*, 2018), a different presentation from P2, which presented hypotonia, developmental delay, learning difficulties and motor regression, nevertheless presenting a typical mitochondrial disease phenotype with presence of lactate peak in spectroscopy and 3-methylglutaconic aciduria.

P3 presented two compound heterozygous missense mutations, c.1268C>T (p.Thr423Met), also found in P2, and c.766C>T (p.Arg256Cys) segregating from the father with a clinical history of infant seizures and exercise intolerance with frequent headaches in adulthood. An association of headaches and exercise intolerance was found for the paternal family carriers of c.766C>T, which was statistically significant. Arg256 residue was highly conserved in bilaterally symmetrical metazoans and was localized in the tip of the hydrophilic arm of NDUFV1 subunit, which unfortunately, was not structurally elucidated and further studies are needed for its functional characterization. Despite that c.766C>T (p.Arg256Cys) is found described in ClinVar as likely pathogenic and, the Arg257 residue, neighboring Arg256, was associated with CI deficiency (Nafisinia *et al.*, 2016). This residue was shown to be essential for protein function and predicted to be post-translationally modified to a N-methylarginine by similarity with the mouse protein (Fang *et al.*, 2017). The variant c.766C>T (p.Arg256Cys) was predicted as pathogenic (score 0.99 and 0.87) and we propose that in combination with c.1268C>T (p.Thr423Met), they might be responsible for P3 phenotype, characterized by a childhood-onset development of hypotonia, muscle weakness, physical exercise intolerance, psychomotor regression, lethargy, dysphagia, and strabismus. The same phenotype was commonly associated with mild CI deficiency (Fassone and Rahman, 2012; Hirst, 2013; Baertling *et al.*, 2018). This phenotype could be explained by a dominant-negative effect of the post-translational modification in the affected region, masking the effect of the wild-type version of the protein. 

## Conclusion

CI deficiency was associated with many nuclear and mitochondrial genes, and the understanding of the genetics of this mitochondrial disease has expanded significantly in recent years. Here we described three cases of mutations in the NDUFV1 subunit, associated with mitochondrial disease and possibly CI deficiency. The case reports and the review of *NDUFV1* mutations showed heterogeneous phenotypes and severity. We propose the association of p.Arg256Cys to frequent headaches in family members of P3. Combination of p.Thr423Met and p.Arg256Cys mutations in heterozygosity were sufficient to cause the clinical features of an autosomal recessive CI deficiency, but confirmation of this deficiency by biochemical approaches were not possible and further studies are necessary.

The addition of new mutations to the literature contributes to a better understanding of the etiology of mitochondrial disease, as well as the potential of future correlations between genotype and phenotype, allowing the earlier implementation of mitochondrial dysfunction preventive therapies.
